# Anatomical Variations in the Branching Pattern of the Left Coronary Artery: A Cadaveric Study in the Eastern Indian Population

**DOI:** 10.7759/cureus.108074

**Published:** 2026-04-30

**Authors:** Gyanaranjan Nayak, Niranjan Sahoo, Dhiren K Panda, Gyanraj Singh, Sujita Pradhan

**Affiliations:** 1 Anatomy, IMS and SUM Hospital, Siksha 'O' Anusandhan (Deemed to be University), Bhubaneswar, IND; 2 Forensic Medicine, All India Institute of Medical Sciences, Bhopal, Bhopal, IND; 3 Anatomy, Maharaja Jajati Keshari Medical College and Hospital, Jajpur, IND

**Keywords:** branching pattern, cadaveric study, coronary artery bypass grafting (cabg), coronary artery disease, left coronary artery

## Abstract

Background: Knowledge of the anatomical variations of the left coronary artery (LCA) is essential for accurate interpretation of coronary angiography, interventional planning, and surgical revascularization. Branching pattern variability directly influences myocardial perfusion and the extent of ischemic injury. This study aimed to document the branching pattern of the LCA in adult cadaveric hearts from Eastern India.

Methods: Sixty formalin-fixed adult human cadaveric hearts were dissected in the Department of Anatomy, IMS and SUM Hospital, Siksha ‘O’ Anusandhan (Deemed to be University), Bhubaneswar, India. The origin of the LCA from the left aortic sinus was identified, and the epicardial fat in the left atrioventricular groove and anterior interventricular sulcus was removed using blunt dissection to expose the main trunk of the LCA and its major branches, like the left anterior descending (LAD) and left circumflex artery (LCx), following them distally. The LCA was thus exposed from its origin to termination, and its branching pattern was classified into bifurcation, trifurcation, tetrafurcation, and any other variations. Of course, branching or termination of major branches of the LCA or branches from such major branches was also noted. All findings were photographed, documented, and quantitatively analyzed.

Results: A total of 60 formalin-fixed adult human cadaveric hearts were examined. Bifurcation, in which the LCA divided into the LAD and LCx arteries, was the most common configuration and was observed in 42/60 hearts (70%). In the bifurcation pattern, the branches were separate proximal to the terminal division. Trifurcation, in which the LCA gave rise to the LAD, LCx, and ramus intermedius, was seen in 15/60 hearts (25%). Tetrafurcation, characterized by division into the LAD, LCx, and two rami intermedii, was present in 3/60 hearts (5%). The 95% confidence Interval (CI) for bifurcation was 58.4 to 81.6%, for trifurcation, 95% CI was 14.1% to 35.9 %, and for tetrafurcation, 95% CI was 0% to 10.6 %.In addition, the LCA terminated as the left marginal artery (included in the category of bifurcation of LCA) in two of 60 hearts, while the left marginal artery arose directly from the LCA (included in the category of trifurcation of LCA) in another 2/60 hearts.

Conclusion: Bifurcation was the most common LCA branching pattern. Some rare variations (not reported in research papers yet from Eastern India) involving the left marginal artery were also noted in the study. Detailed anatomical knowledge of LCA variation assists clinicians in improving diagnostic accuracy and enhancing the safety of interventional and surgical procedures. However, our study has some limitations, such as a relatively small sample size and a single-region cadaveric study. So we propose larger multicentric radiological and interventional studies, which will be of great benefit to the surgical and radiological community.

## Introduction

The coronary arterial system supplies oxygenated blood to the myocardium and is essential for normal cardiac function. The two main coronary arteries arise from the ascending aorta, with the left coronary artery (LCA) typically originating from the left posterior aortic sinus and supplying a larger portion of the myocardium than the right coronary artery (RCA). The LCA usually divides into the left anterior descending (LAD) and left circumflex (LCx) arteries, although significant variation exists in its branching pattern, including bifurcation, trifurcation, tetrafurcation, and higher-order divisions [1,2].

Previous anatomical studies report a wide spectrum of morphological variability in LCA branching. Bifurcation is generally the most common pattern, with frequencies ranging between 45% and 80% [3]. Trifurcation rates vary from 13% to 42% across populations [3-4]. While tetrafurcation and pentafurcation have been documented less frequently, typically between 1% and 11% [3, 4]. These variations influence coronary blood flow patterns and may influence the distribution of atherosclerotic lesions or complicate interventional procedures such as angiography and coronary artery bypass grafting (CABG) [1].

The embryogenesis of the LCA involves primordial origin from the proepicardial organ, sprouting of vessels from the primordium, the subsequent fusion of these sprouts with the aortic root, and establishment of the definitive LCA orifice, followed by triggering of the earlier sprouted vessels getting remodeled into LCA, LAD, and LCx branches. The variations of branching pattern may be attributed to errors in the formation of sprouts and their positioning and their subsequent reconnection with the aorta [1-4].

Hemodynamic complications of the variable branching pattern of the LCA include altered wall shear stress leading to atherosusceptibility (due to the trifurcation of the LCA) and redistributing blood from the LCA to the marginal branch (due to the ramus intermedius), resulting in ischemia in selected areas [1-4].

Understanding coronary variations is essential for cardiologists, cardiothoracic-vascular surgeons, and radiologists, particularly in diagnostic imaging, angiographic interpretation, and planning of revascularization procedures. Because coronary patterns may vary significantly among populations, regional cadaveric studies remain critical for improving clinical accuracy and enhancing anatomical knowledge.

Although variations in the branching pattern of the LCA are well documented, population-specific anatomical data from Eastern India (particularly Odisha and some of its neighboring eastern states of India) are limited. In fact, to our knowledge, no such study has been reported from the state of Odisha to date. Additionally, certain rare variations of the LCx artery and left marginal artery are not highlighted in research papers on variations of the LCA originating from Eastern India, highlighting the need for further cadaverous investigation in this population.

## Materials and methods

This descriptive cadaveric study was conducted in the Department of Anatomy, IMS and SUM Hospital, Siksha ‘O’ Anusandhan (Deemed to be University), in Bhubaneswar, Odisha, India, using 60 formalin-fixed adult human cadaveric hearts. The specimens were preserved in the Departmental Museum of Anatomy. They belonged to adults of the age group 50-70 years of both sexes (40 males and 20 females), dying of natural causes. Specimens with intact coronary anatomy were included, while hearts with traumatic, pathological, or iatrogenic damage to the coronary vessels were excluded.

Epicardial fat was carefully dissected in all the preserved hearts to expose the coronary arteries following the technique described by Ravi and Tejesh (2017) [3] and Alam [5]. The LCA was identified at its origin from the left posterior aortic sinus and traced until its termination.

Classification of the branching pattern

The branching pattern of the LCA was categorized according to the number of terminal branches arising from its main trunk. Bifurcation was defined as the division of the LCA into the LAD and LCx arteries, trifurcation as division into the LAD, LCx, and ramus intermedius, and tetrafurcation as division into the LAD, LCx, and two rami intermedii. Any other abnormalities in the branching pattern of the LCA were also noted. Assessment of coronary dominance was excluded from the study. Ethical clearance was not required, as the study was conducted on specimens preserved in the Departmental Museum of Anatomy of a medical college, and these specimens had been obtained from human cadavers dissected during routine MBBS dissection classes. The course and termination of the LCA and its branches were carefully documented, and all observations were photographed for record and analysis.

The authors have not included the anomalous origin of the posterior interventricular artery, as they have already studied it in one of their earlier published works [6].

Statistical analysis

All data were entered into Microsoft Excel (Microsoft Corp., Redmond, WA, USA) and analyzed descriptively. Frequencies and percentages were calculated for each branching pattern (bifurcation, trifurcation, tetrafurcation) and other anomalies. A 95% confidence Interval (CI) was calculated. This analytical approach aligns with similar cadaveric coronary studies [1, 2].

## Results

A total of 60 formalin-fixed adult human cadaveric hearts were examined. The LCA was successfully identified in all specimens. Observations related to branching pattern and ancillary anatomical features are presented below.

Variations of the branching pattern of the LCA

Three distinct branching patterns of the LCA were identified in this study. The distribution of these patterns is presented in Table 1.

**Table 1:** Variations of left coronary artery (LCA) branching patterns (n = 60)

Variation of the LCA	n (%)
Bifurcation of the LCA	42 (70%)
Trifurcation of the LCA	15 (25%)
Tetrafurcation of the LCA	3 (5%)

**Figure 1:**
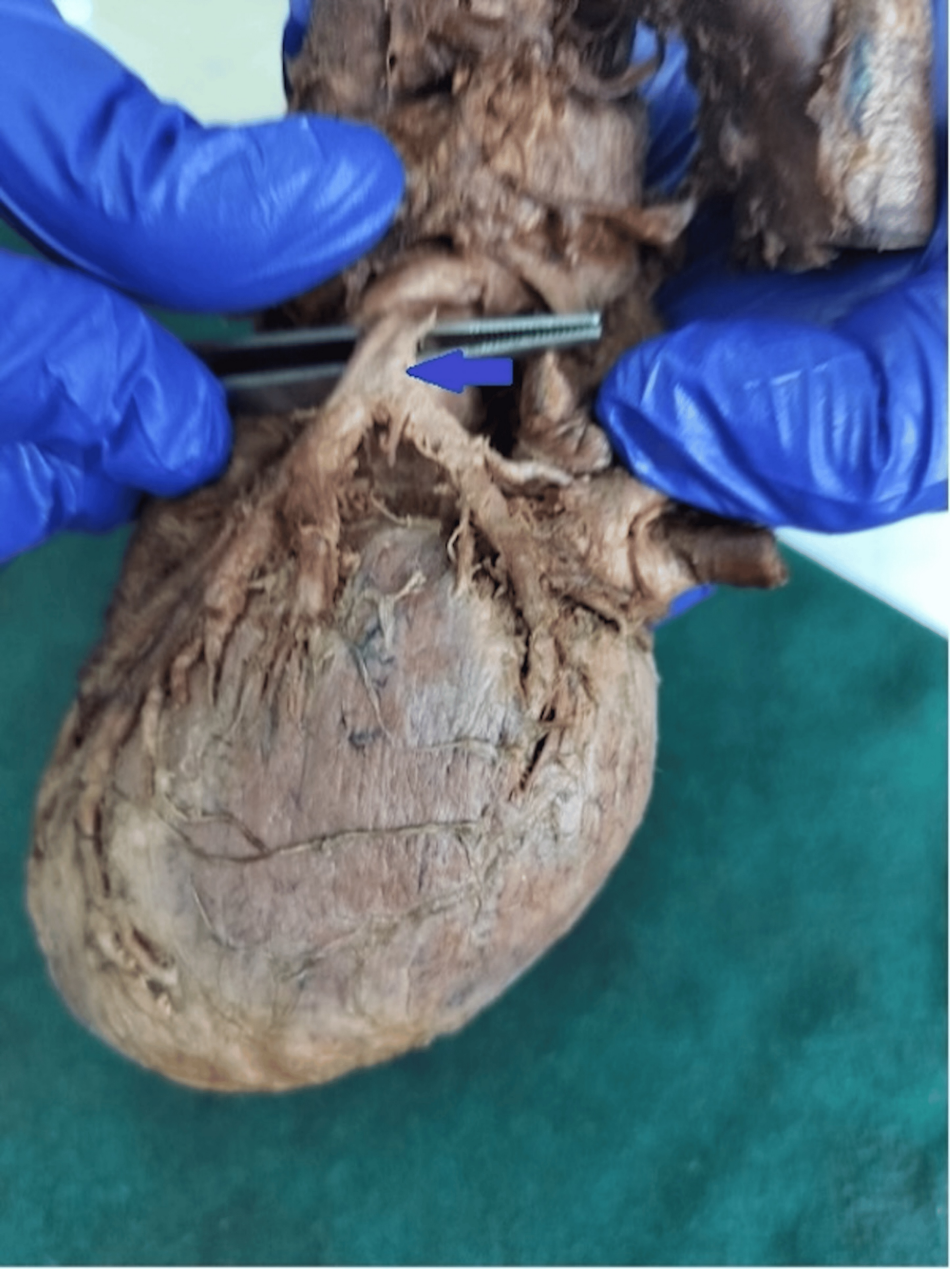
Cadaveric heart specimen showing bifurcation of the left coronary artery (LCA); the arrow indicates the vessel of interest. The LCA trunk is elevated with forceps, demonstrating its division into the left anterior descending (LAD) artery descending in the anterior interventricular sulcus and the left circumflex (LCx) artery coursing toward the atrioventricular groove.

Bifurcation, in which the LCA was divided into the LAD and LCx arteries, was the most common configuration, observed in 42 hearts (70%) (Figure 1). 

**Figure 2:**
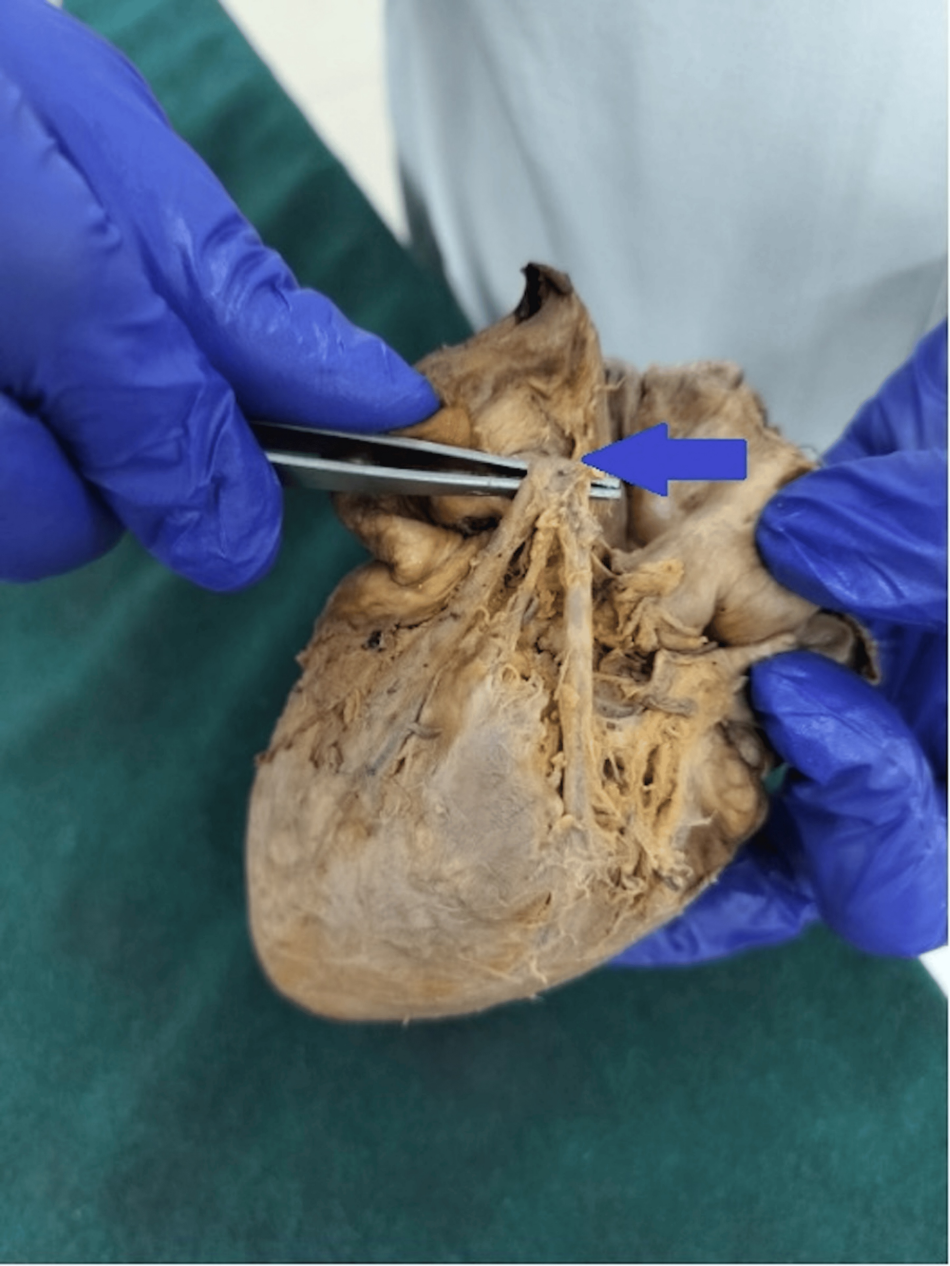
Cadaveric heart specimen demonstrating trifurcation of the left coronary artery (LCA); the arrow indicates the vessel of interest. The LCA trunk is elevated with forceps, showing its division into the left anterior descending (LAD) artery, left circumflex (LCx) artery, and an additional ramus intermedius branch arising between them.

Trifurcation, with the LCA giving rise to the LAD and LCx arteries, and ramus intermedius, occurred in 15 specimens (25%) (Figure 2). 

**Figure 3:**
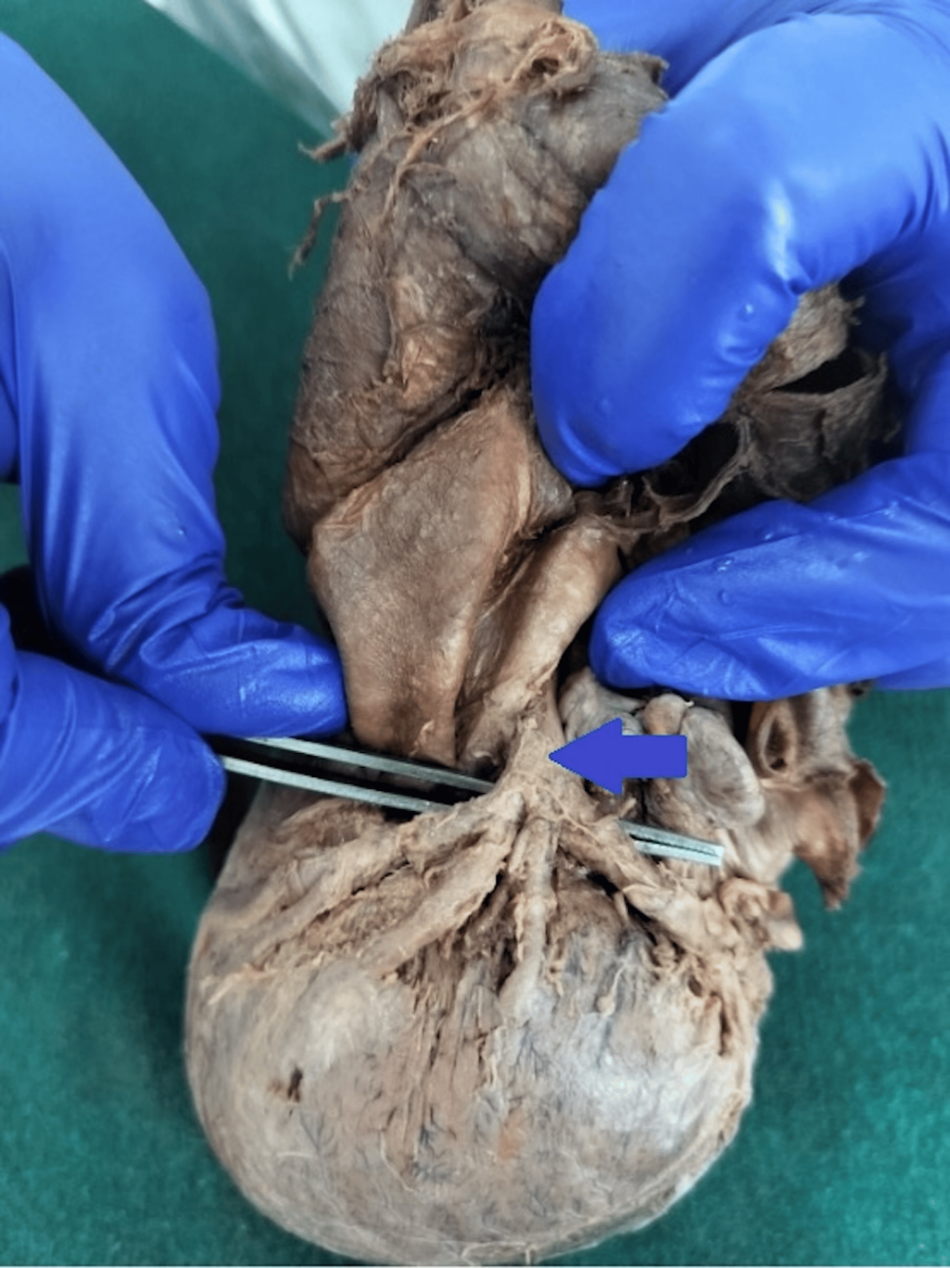
Cadaveric heart specimen showing tetrafurcation of the left coronary artery (LCA); arrow indicates the vessel of interest. The LCA trunk is elevated with forceps, demonstrating division into the left anterior descending (LAD) artery, left circumflex (LCx) artery, and two ramus intermedii branches originating directly from the main LCA trunk.

Tetrafurcation, characterized by two rami intermedii branches arising in addition to the LAD and LCx arteries, was noted in three specimens (5%) (Figure 3). 

A CI of 95% was calculated and found as follows: for bifurcation of LCA, it was 58.4% to 81.6%, for trifurcation of LCA, it was 14.1% to 35.9%, and for tetrafurcation of LCA, it is 0% to 10.6%.

**Figure 4:**
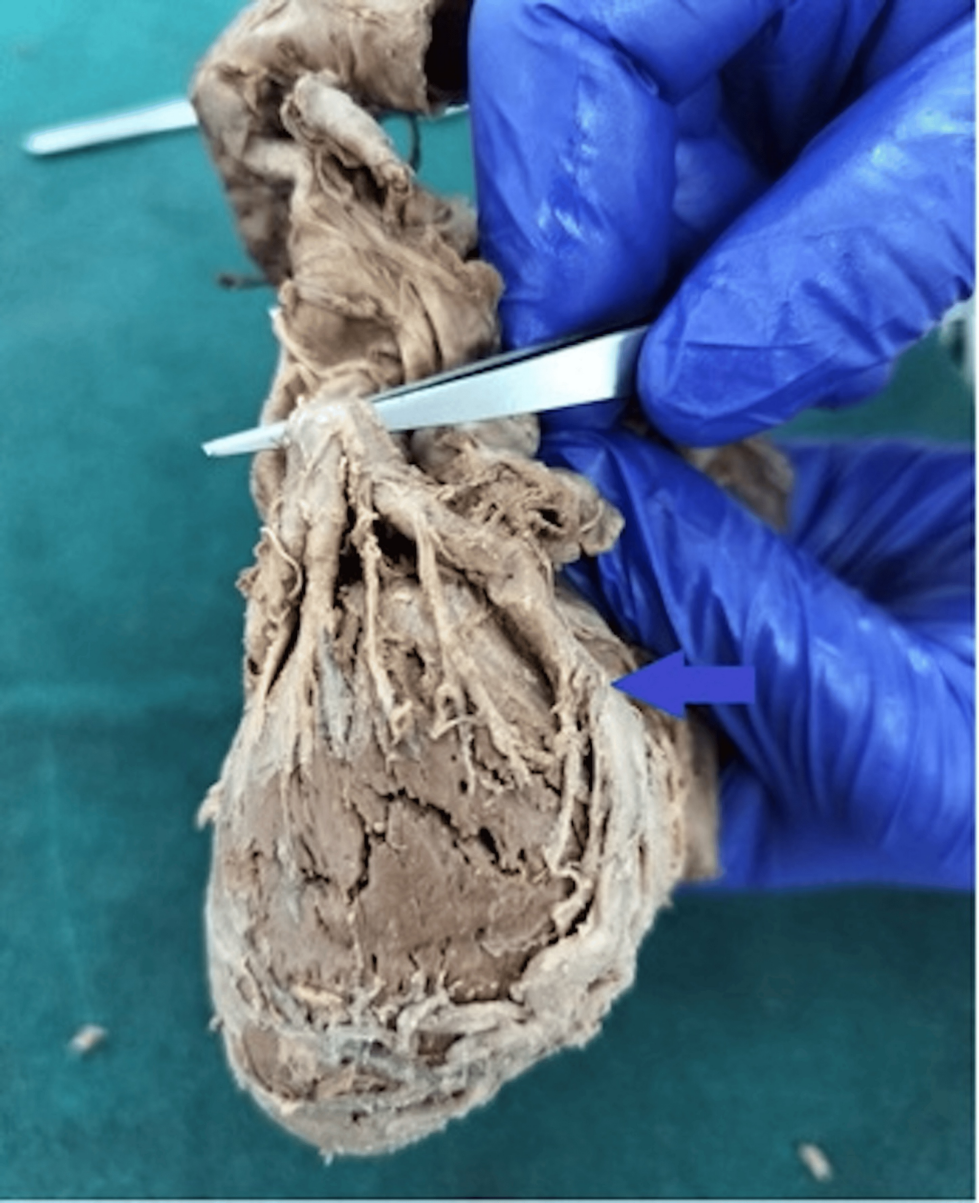
The LCx left circumflex terminating as the left marginal artery; the arrow indicates anomalous course and termination.

Figure 4 shows the LCx artery terminating as the left marginal artery (LCA bifurcated) in two out of 60 specimens.

**Figure 5:**
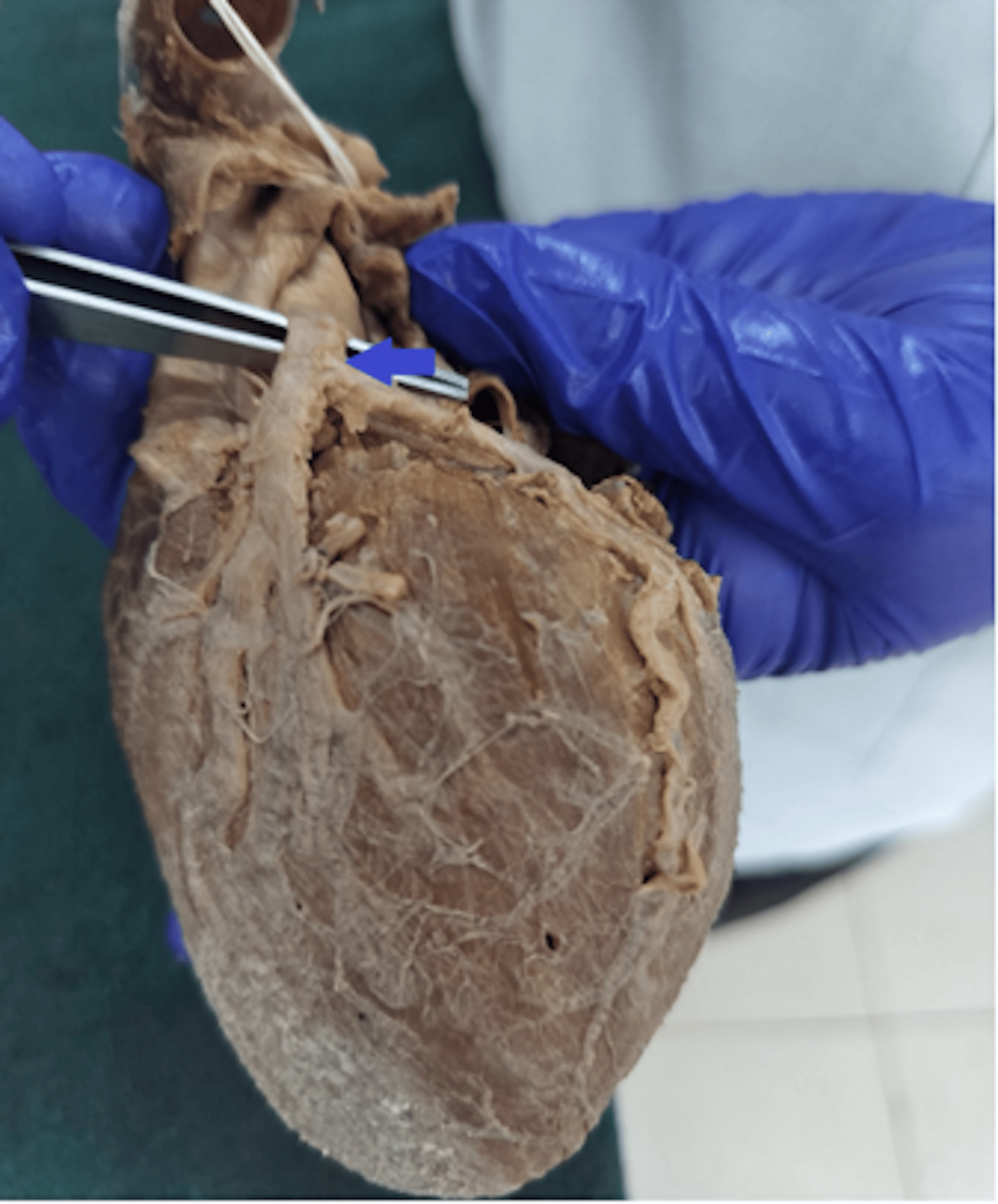
Left marginal artery arising directly from the left coronary artery (LCA); the arrow indicates the anomalous origin.

Figure 5 shows the left marginal artery arising from the LCA (LCA trifurcated) in two out of 60 specimens.

Additional observations

The LCA could be traced from its origin to its termination in all specimens. No coronary anomalies such as single coronary artery, absent LCA trunk, separate LAD/LCx ostia, or anomalous origins were identified. No myocardial bridges or coronary fistulae were observed in any specimens.

## Discussion

The present cadaveric study demonstrates considerable variability in the branching pattern and dominance of the LCA in an Eastern Indian population. Bifurcation was the predominant pattern (70%), consistent with findings from Ravi & Tejesh (2017), who also identified bifurcation as the most common terminal pattern [3]. Trifurcation (25%) and tetrafurcation (5%) frequencies in the present study also align with previous anatomical research reporting comparable variation in LCA branching patterns [2-4].

To contextualize these observations, Table 2 provides a comparative overview of LCA branching patterns reported in nine anatomical studies, including the present series. As seen, bifurcation is universally the most common pattern, while higher-order divisions (tetrafurcation/pentafurcation) show wide population variability.

**Table 2:** Comparison of left coronary artery (LCA) branching patterns across anatomical studies

Study	Number	Bifurcation (%)	Trifurcation (%)	Tetrafurcation (%)	Pentafurcation (%)
Present study	60	70	25	5	0
Lakshmiprabha et al. [1]	55	54.54	41.82	1.82	1.82
Ravi & Tejesh [3]	30	80	13.13	3.3	3.3
Malashetty & Itagi [4]	30	66.67	23.33	10	0
Alam [5]	50	74	26	0	0
Yassa et al. [7]	430	77	14	9	0
Bhavya et al. [8]	53	45.3	45.3	5.7	3.8
Bharadwaj et al. [9]	80	45	30	12.5	0
Al-Omari et al. [10]	212	78.3	22.7	0	0
Anandi et al. [11]	62	58	35.4	6.4	0
Dharmendra et al. [12]	93	58.06	35.48	6.45	0
Beger et al. [13]		68.77	26.77	1.01	<0.01

Assessment of the abnormal origin of the posterior interventricular artery from the LCA was excluded from the present study, as the authors had already addressed coronary dominance in an earlier published work [6]. Moreover, the abnormal origin of the posterior interventricular artery is more appropriately considered an anomaly of the LCx rather than a direct variation of the LCA itself. However, we hereby put forward that in our previous study on the abnormal origin of the posterior interventricular artery (n=60), we got the following results: right coronary predominance in 42 (70%) cases, left coronary predominance in six (10%) cases, and coronary codominance in 12 (20%) cases [6]. Variations in the branching pattern of the left main coronary artery may have important implications for CABG planning and surgical outcomes [13, 14]. The presence of additional branches, such as a ramus intermedius in trifurcation or tetrafurcation, requires precise preoperative imaging, including CT coronary angiography, to identify the target vessels requiring grafting. Such variations may also influence graft selection, graft length, and conduit choice, whether arterial or venous, depending on the course and distribution of the vessels. In addition, abnormal branching patterns can make the planned surgical anastomosis technically more challenging. Failure to identify these anatomical variations preoperatively may result in incomplete revascularization and inadequate myocardial perfusion through the newly established grafts.

This study provides region-specific cadaveric data on the branching pattern of the LCA in the Eastern Indian population and highlights uncommon variations involving the LCx artery and left marginal artery (yet not reported by any research paper from Eastern India, to our knowledge), contributing additional anatomical evidence relevant to coronary imaging and surgical planning. But a relatively small sample size in the current study calls for more detailed research on a larger scale.

Overall, our results contribute valuable region-specific data on coronary anatomy. The presence of typical and atypical branching patterns underscores the importance of careful preoperative and angiographic evaluation, especially in populations where anatomical variability may differ significantly from global averages. Such knowledge is not only essential for anatomical understanding but also for optimizing interventional cardiology and cardiac surgical outcomes [14].

This study is limited by its relatively small sample size, single-region cadaveric design, and absence of radiological or clinical correlation; therefore, the findings should be interpreted as descriptive anatomical observations that would benefit from confirmation in larger multicentric studies.

## Conclusions

The current study highlights that bifurcation is the most common branching pattern of the LCA, which is in accordance with most of the available literature. The study also reports various variations of the branching pattern of the LCA and other alterations of the LCx and left marginal artery. The variations described by us in the Eastern Indian population can result in changes in coronary flow and other hemodynamic effects influencing the distribution of coronary atheromatous plaques, as evidenced by various articles cited in the study. These variations can have a significant influence on planning and performing radiological or surgical coronary interventions. So it is advisable to keep these variations in mind because if ignored, they may have adverse outcomes while performing angiography or CABG.
